# Interventions targeting hypertension and diabetes mellitus at community and primary healthcare level in low- and middle-income countries:a scoping review

**DOI:** 10.1186/s12889-019-7842-6

**Published:** 2019-11-21

**Authors:** Jorge César Correia, Sarah Lachat, Grégoire Lagger, François Chappuis, Alain Golay, David Beran, On behalf of the COHESION Project, David Beran, Jaime Miranda, Albertino Damasceno, Claire Somerville, L. Suzanne Suggs

**Affiliations:** 1 Division of Tropical and Humanitarian Medicine, Department of Community Medicine, Primary and Emergency Care, Geneva University Hospitals and University of Geneva, 1205 Geneva, Switzerland; 2 Division of Therapeutic Patient Education for Chronic Diseases. Department of Community Medicine, Primary and Emergency Care, Geneva University Hospitals and University of Geneva, 1205 Geneva, Switzerland

**Keywords:** non-communicable diseases, diabetes, hypertension, primary health care, low- and middle-income countries

## Abstract

**Background:**

Hypertension (HTN) and diabetes mellitus (DM) are highly prevalent in low- and middle-income countries (LMIC) and a leading cause of morbidity and mortality. Recent evidence on effectiveness of primary care interventions has attracted renewed calls for their implementation. This review aims to synthesize evidence pertaining to primary care interventions on these two diseases, evaluated and tested in LMICs.

**Methods:**

Two reviewers conducted an electronic search of three databases (Pubmed, EMBASE and Web of Science) and screened for eligible articles. Interventions covering health promotion, prevention, treatment, or rehabilitation activities at the PHC or community level were included. Studies published in English, French, Portuguese and Spanish, from January 2007 to January 2017, were included. Key extraction variables included the 12 criteria identified by the Template for Intervention Description and Replication (TIDieR) checklist and guide. The Innovative Care for Chronic Conditions Framework (ICCCF) was used to guide analysis and reporting of results.

**Results:**

198 articles were analyzed. The strategies focused on healthcare service organization (76.5%), community level (9.7 %), creating a positive policy environment (3.6%) and strategies covering multiple domains (10.2%). Studies included related to the following topics: description or testing of interventions (n=81; 41.3%), implementation or evaluation projects (n=42; 21.4%), quality improvement initiatives (n=15; 7.7%), screening and prevention efforts (n=26; 13.2%), management of HTN or DM (n=13; 6.6%), integrated health services (n=10; 5.1%), knowledge and attitude surveys (n=5; 2.5%), cost-effective lab tests (n=2; 1%) and policy making efforts (n=2; 1%). Most studies reported interventions by non-specialists (n=86; 43.4%) and multidisciplinary teams (n=49; 25.5%).

**Conclusion:**

Only 198 articles were found over a 10 year period which demonstrates the limited published research on highly prevalent diseases in LMIC. This review shows the variety and complexity of approaches that have been tested to address HTN and DM in LMICs and highlights the elements of interventions needed to be addressed in order to strengthen delivery of care. Most studies reported little information regarding implementation processes to allow replication. Given the need for multi-component complex interventions, study designs and evaluation techniques will need to be adapted by including process evaluations versus simply effectiveness or outcome evaluations.

## Background

Non-communicable diseases (NCDs) are global public health concerns, with four conditions receiving a priority status by the World Health Organization: cardiovascular diseases (CVD), chronic respiratory diseases, diabetes mellitus (DM) and cancers [1]. Described as the “invisible epidemic” [2], NCD mortality exceeds that of communicable, maternal, perinatal and nutritional conditions combined [3, 4]. NCDs are the largest cause of mortality both globally and in the majority of low- and middle- income countries (LMICs) [5–8] where approximately 80% of the global deaths from NCDs occur [9].

Management of NCDs requires regular availability of drugs, laboratory facilities, data collection tools, trained healthcare workers and educated and empowered patients in addition to health services tailored to the social and life characteristics of individuals [10–12].

There is strong evidence that primary care is one of the most cost-effective strategies in curbing morbidity, disability and premature mortality of hypertension (HTN) and DM [13, 14]. The need for effective primary care interventions was stated in the Alma Ata Declaration in 1978, which emphasized effective healthcare systems as a reflection of social determinants rather than hospitals and doctors alone [15]. The Declaration proposed a focus on Primary Health Care (PHC) which challenged the view of biomedicine dominated healthcare system [15]. PHC conceptualized healthcare as scientific, socially acceptable and universally accessible and based on the principles of equity and community participation [15]. PHC has again been in the spotlight with the 40-year anniversary of the Alma-Ata Declaration and the global community reasserting its principles in the Astana Declaration, which emphasized the importance of PHC in achieving universal health coverage and the sustainable development goals, and on the prevention and management of NCDs [15].

Recognizing the importance of PHC, the WHO has developed the Package of Essential Non-communicable Disease Interventions (WHO PEN) for Primary Care in low-resource settings [13, 14].. The WHO PEN has a special focus on hypertension (HTN) and DM and their integrated management given their burden. Research and policy making efforts are underway in many countries, however, there are no scoping reviews or evidence synthesis efforts related to interventions targeting HTN and DM in LMICs. To address this paucity of data, we conducted a scoping review focusing on these two diseases. This review aims to describe the key characteristics of HTN and DM focused primary care and community level interventions in LMICs [16].

## Methods

This review was guided by the framework for scoping reviews recommended by Arksey and O’Malley [17]. As opposed to systematic reviews, this approach was found to be more appropriate for mapping key concepts in this vast research area, spanning across heterogeneous domains and disciplines [18, 19].

Using a pre-defined search strategy, two reviewers conducted a search in Pubmed, EMBASE and Web of Science. Studies published in English, French, Portuguese and Spanish, from January 2007 to January 2017, were included (see Additional file 1 for the complete search strategies). The results of all searches were entered into the Covidence software for analyses [20]. After duplicates were removed, the remaining citations were assessed by title and abstract and then by full texts. The two reviewers independently assessed articles for eligibility against the study inclusion criteria. Disagreements about the inclusion of studies were resolved through discussion and consensus. Bibliographies of eligible full texts were also examined for potentially relevant articles based on the eligibility criteria.

All studies were judged on following criteria for inclusion:

- Interventions developed for populations affected by DM and HTN in LMICs, as defined by the World Bank [21].
- Interventions covering health promotion, prevention, treatment, or rehabilitation activities at the PHC or community level.
- Interventions focusing on health system organization, policy making, financing of health care systems.
- Community level intervention was defined as any intervention delivered at home, village, or any defined community setting, but not in a health facility.
- All those studies that reported prevalence or other cross-sectional descriptions of health system or populations in LMIC were excluded.

Key extraction variables included the 12 criteria identified by the *Template for Intervention Description and Replication* (TIDieR) checklist and guide [22]. This included several variables related to implementation processes such as a) the name of the intervention b) rationale of intervention c) materials and procedures d) delivery agent of intervention e) density of dosage of intervention f) flaws in study design g) training, and supervision of delivery agents h) fidelity rating h) manualization or tailoring of interventions. The Innovative Care for Chronic Conditions Framework (ICCCF) [23] was used as the analytical framework to guide analysis and reporting of results. The ICCCF is based on the well-known Chronic Care Model and highlights the importance of the policy, health system, community and individuals in providing the environment necessary for improving chronic patient care [24–27]. For the purpose of this review, the ICCCF framework was used to classify interventions at three levels: a) healthcare service organization b) Community level and c) Policy making. These levels were further sub-classified into several strategies. Healthcare service organizational level strategies spanned across: self-management through education and monitoring, continuity and coordination, information systems, leadership incentives and organizing and equipping healthcare teams. Community level interventions were sub-classified into: a) mobilization of communities to participate b) raising awareness c) provision of complementary services and leadership support. Lastly, policy level initiatives included: a) integration of policies b) supportive legislations c) human resource d) strengthening partnerships e) leadership and advocacy f) consistent financing. Interventions spanning across several strategies within the same level were labelled as multifaceted. Those interventions that employed strategies from different levels were categorized as “multiple domains”.

## Results

### Characteristics of studies

**Fig. 1:**
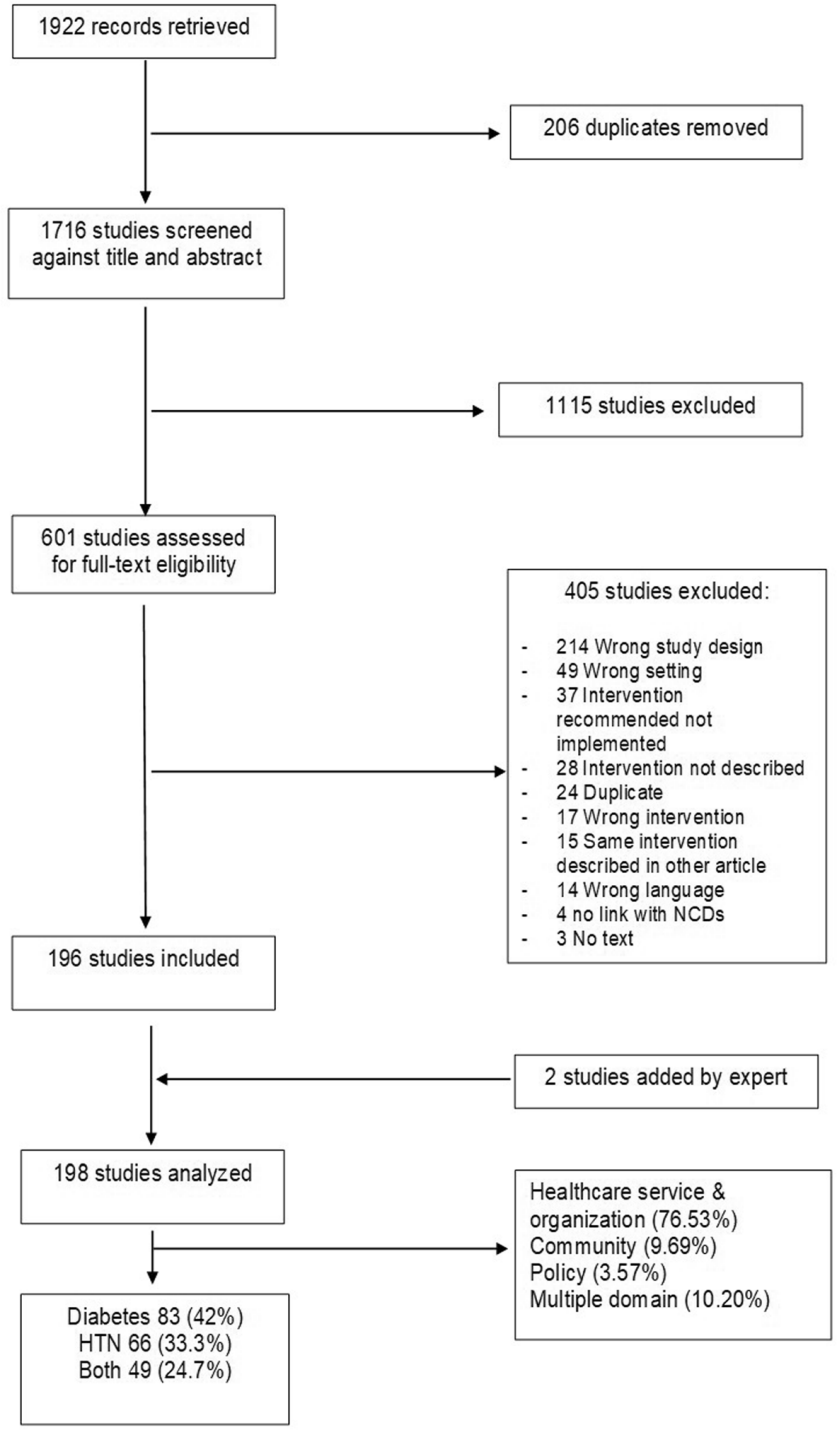
Flow chart for the articles included in the scoping review

The initial search identified a total of 1922 citations. After duplicates were removed, 1716 were accepted for further screening. A total of 1115 articles did not fulfill the inclusion criteria and thus, were excluded. A total of 601 studies were accepted for full text review. Of these, 196 studies were identified as meeting the inclusion criteria. Most of the studies were excluded because of study design such as prevalence studies (n=214), not from the LMIC (n=49), recommended intervention not implemented at the PHC or community level (n=37), and studies that lacked details on the content of intervention (n=28). Two additional articles were added as per suggestions by a subject expert. The flow chart for the articles included in the scoping review are described in Fig. 1. A total of 83 papers were related to DM, 66 to HTN, and 49 to both. Papers included described a variety of populations. The largest sample size was reported as 25,000 Turkish schools, reaching over 7.5 million students and 600,000 teachers [28]. Papers were from 43 countries, most commonly Brazil (n=31), China (n=26), Thailand (n=20), Mexico (n=13) and South Africa (n=13). The characteristics of the different studies are detailed in Additional file 2.

**Fig. 2:**
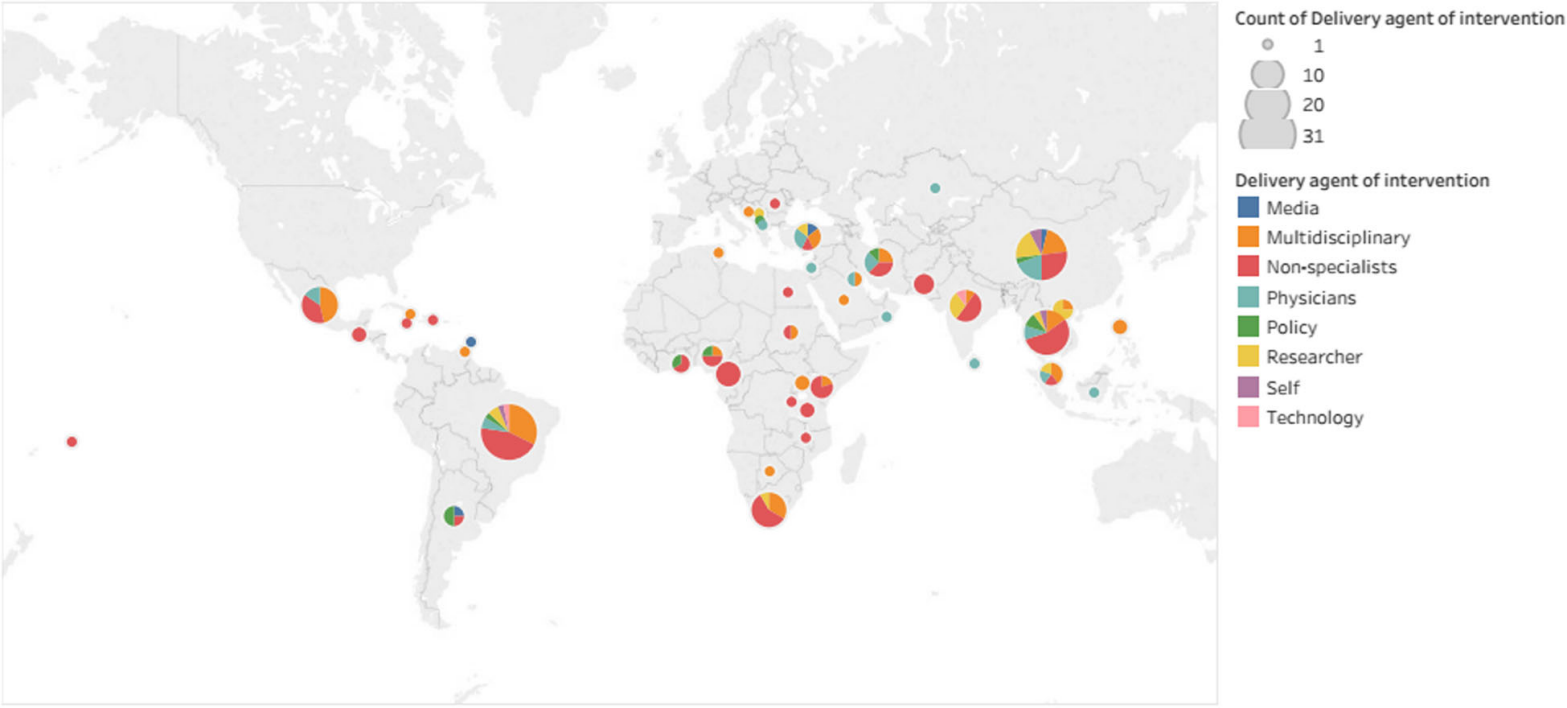
Characteristics of included studies according to the ICCF domain

All studies were assessed according to the ICCCF framework, to delineate the dominant strategies tested in the interventions. This framework provided a reproducible approach to classify the interventions into four domains: health service organization, community initiatives, policy making and those packing strategies from multiple domains. Most of the strategies focused on healthcare service organization (76.3%), followed by community level interventions (9.6 %) and finally creation of a positive policy environment (3.5%). Furthermore, there are strategies covering multiple domains (10.7%). The characteristics of included studies according to the ICCF domain are represented in Fig. 2. Further description of the included studies according to the TIDieR template and their classification according to the ICCF are shown in Additional file 3.

Study designs were descriptive i.e. reporting post-intervention outcomes in descriptive manner (n=81; 40.91%), RCTs (n = 42 ; 21.21%), quasi-experimental with pre-post design (n=28; 14.14%), cluster RCT (n=16; 8.08%), longitudinal (n=16; 8.08%), cross-sectional (n=7; 3.54%), and others including mixed-methods, qualitative, operational and case-studies (n= 8; 4.04%). Primary aims of the studies were description or testing of an intervention 82 (41.41%), implementation or evaluation projects (n=43; 21.72%), quality improvement initiatives (n=15; 7.58%), screening and prevention efforts (n=26; 13.13%), clinical management of HTN or DM (n=13; 6.57%), integrated health services (n=10 ; 5.05%), knowledge and attitude surveys (n=5; 2.53%), cost-effective lab tests (n=2; 1.01%) and policy making efforts (n=2; 1.01%). A majority of the studies (n=166; 84.69%) reported interventions integrated in primary care hospitals, clinics and pharmacies, while others (n=32; 16.16%) were non-integrated into healthcare settings. Most of the studies were conducted in urban areas (n=124; 60.63%) followed by rural areas (n=15; 7.58%), national level (n=11; 5.55%), provincial (n=4; 2.02%), while only few (n=5; 2.53%) studies reported findings in multiple countries. Only 5 studies reported the intervention to be conducted in special settings: poor parishes, indigenous populations (n=1, 0.51%) or conflict areas (n=1; 0.51%).

### Intervention delivery

**Fig. 3:**
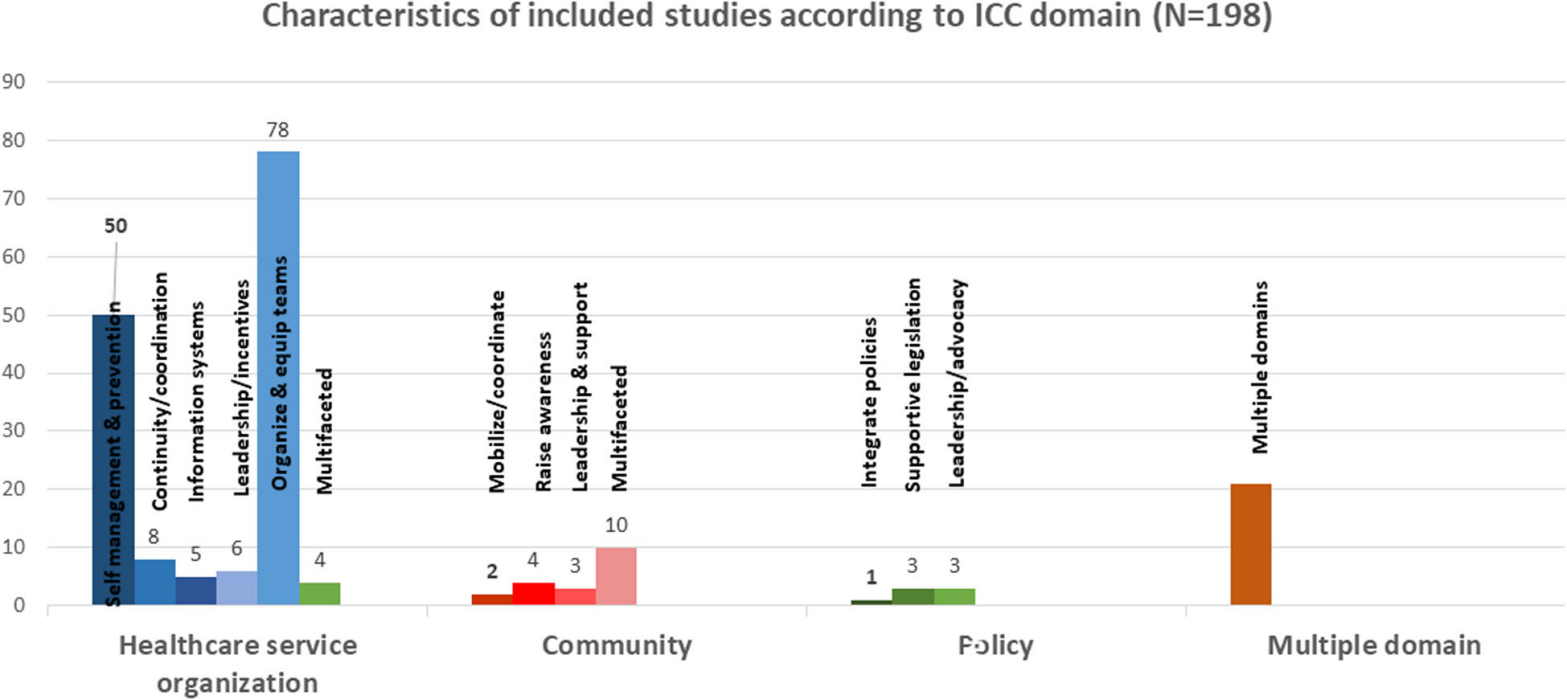
Delivery agents involved in included studies. This figure was created using the Tableau Desktop software version 2018.3.8 licensed for noncommercial academic research

These studies reported varied types of personnel delivering interventions. A high proportion of studies reported interventions by non-physicians (n=86; 43.43%), followed by multidisciplinary teams including physicians as well as non-specialists (n=49; 24.75%), physicians (n=23; 11.62%), researchers (n=18; 9.09%), policy makers (n=11; 5.56%), media (n=4; 2.02%), patients (n=4; 2.02%) and technology-based interventions (n=3; 1.52%). Among non-physician delivered programs, a majority were delivered by nurses (n=23), followed by community health workers (CHW) (n=18), pharmacists (n=14), nutritionists and dieticians (n=9), peers (n=7), counsellors & educators (n=4), physical trainers (n=3), medical assistants, technicians and non-physicians (n=3), and finally students (n=4). The count and delivery agents involved in included studies are represented in Fig. 3.

A majority of the studies did not mention explicit training methods for delivery agents (n=138). When this information was available, delivery agents were trained by researchers (n= 32), physicians (n= 7), nurses (n= 5), specialist physicians (n= 4), certified educators (n= 3), multidisciplinary teams (n= 2), and trainer (n= 1), accrediting body (n= 1), foreign collaborators (n= 1), physical trainers (n= 1), and nutritionist (n= 1).

### Healthcare System Organization

The majority of papers (n=151) focused on strategies at the level of healthcare service organization. A high number of studies focused on organization and equipping healthcare (n= 78), followed by self-management by education and self-monitoring (n= 50), continuity and coordination (n =8), leaderships incentivization (n= 6), use of information systems (n = 5) and multifaceted interventions (n = 4).

### Self-management by education and self-monitoring

A total of 50 papers focused on strategies to support *self -management by education and self-monitoring,* through education or self-monitoring. Educational strategies constituted the primary focus of 46 papers, with a goal to increase knowledge about the disease(s) in order to bring behavior change [29–31], promote lifestyle modifications [32–35], improve awareness, the disease(s) treatment and improve clinical outcomes [35–38].

A total of 21 interventions were based on homogeneous themes of knowledge regarding DM, dietary and exercise habits delivered in groups, individualized and based on home visits, sometimes involving patient families. Three studies also focused on communication skills, problem solving and stress management [35, 39, 40]. Five studies reported educational programs delivered by multidisciplinary teams comprising doctors, nurses, psychologists, psychotherapists, counselors and nutritionists who delivered health coaching face to face and telephone [32, 35, 41–43], while Eik et al [44], tested the effectiveness of Brazilian health guideline based on health coaching by specialized instructor and follow ups by an endocrinologist. Two of the studies explored educational interventions delivered by nurses to improve physical activity by delivering exercise referrals and PA counselling intervention [45] while the second intervention focused on a 5 week structured educational module to improve glycemic index [46]. Four studies [47–50] reported educational interventions delivered by nutritionists that aimed to improve adherence to non-pharmacological treatment of HTN, reduce risk factors, and improve dietary behaviors and glycemic control among patients. In contrast to rest of the studies delivering counselling or educational workshops, Ribeiro et al., also implemented family orientations sessions through home visits [50]. Liu et al., ensured good dietary behaviors by delivering pamphlets based on the concept of traffic light diet combined with individualized nutrition counselling every two months after the intervention, over a six months period [47]. Lu et al. [51], sought to improve patient knowledge on HTN using interactive educational workshop while Oliveira et al., emphasized the importance of regular group encounters with educational programs comprising Dietary Approaches to Stop HTN (DASH) diet, physical activity and emphasis on reduction and consumption of alcohol and tobacco [52]. The VIDA project aimed to improve the quality of DM by educating patients about foot care as well as primary care personnel including physicians, nurses, nutritionists and psychologists [53]. Only one educational intervention for management of DM was focused toward a marginalized community, and delivered by a team of non-specialists including nursing students and local health workers over 12 weeks of DM classes and individual follow-ups [54]. Lay facilitators and peer led educational interventions among Thai [39, 40, 55] and Jamaican populations [37], comprising group meetings, individualized sessions as well as home visits. The content of these interventions aimed to improve knowledge regarding DM, self-monitoring dietary habits, group counselling, as well as promoting communication and problem-solving skills.

Lifestyle modification programs were explored in four studies as secondary preventive strategies [33, 34, 38, 56]. These programs were run by nurses in Iran (lifestyle modification package), DM prevention team in Pakistan (lifestyle modification and metformin), and in India and China as DM community lifestyle improvement program offered by professional health educators, exercise trainers and lay-interventionists. Wei et al. [38], implemented a multi-pronged approach delivered as monthly club meetings detailing personalized diet therapy as well as effective communication skills to improve clinical outcomes among patients with DM. Two studies also reported pharmacist led counseling interventions in pharmaceutical set-ups aimed towards lifestyle improvement, and treatment adherence, in collaboration with physicians [57, 58].

Physical activity counseling and/or exercise classes complemented nutritional education in several health promotion programs and were mainly directed for DM prevention or on improvement of glycemic control of diabetic patients in six studies [35, 59–63]. All of these interventions differed in their content and were delivered either by exercise trainer, physician, researcher or the patient themselves. The interventions included long term home based light to moderate intensity walking program spanning three session a week [36], with re-assessment of the participants for BP and anthropometric profile each 2 months [35]. Training in Yogic breathing techniques (sitting, breathing, meditation) was provided to the patients by researchers in a three visit program to improve glycemic index [59]. Other programs included 8 sessions of physical exercise training by a team of physicians [64]. A multi-pronged approach was adopted by Tran et al., which included educational materials on diet, resistance bands for strength exercise and a 6 month membership to a walking group to improve both the dietary and physical activity behaviors [65]. Lastly, Debarros et al [66] tested supervised resistance exercise training to pregnant mothers to improve gestational diabetes*.*

Behavioral techniques were utilized in a total of four studies. Among these interventions, nurses certified in Motivational Interviewing (MI) aimed to counsel HTN patients [67], and Saengtipbovorn et al. [68], evaluated the effectiveness of a multidisciplinary team comprising of doctors, dentists, dental assistants and nurses to improve glycemic index and dental hygiene through MI. Other strategies included counselling of patients to improve depressive symptoms by counsellors [60] and physician delivered smoking cessation counselling [30]. Three studies assessed the effectiveness of empowerment programs delivered by lay health workers in Brazil [29], and multidisciplinary teams comprising a team of nurses, endocrinologist and nutritionists in Iran [61] and Turkey [62]. These programs aimed to improve clinical outcomes and self-management among patients by incorporating behavior change protocols and empowering services.

Four self-management programs focused on nutritional interventions delivered either by nutritionists or physicians in Oman [63], in Mexico [69] and Brazil [70, 71]. These interventions were designed as per Omani practice guidelines, DASH approach adapted for Mexico [69] and a multifaceted program comprising nutrition education, physical activity counselling and community exercise classes (walking and dancing) [70]. Lastly, Lima et al., tested the effectiveness of Brazilian Dietary Approach to Break HTN (BRADA) for reducing glycemic and lipid profiles among hypertensive patients. This diet was also based on DASH with low sodium and low glycemic index foods [71].

Only four papers focused on Self–Monitoring (SM). The Self-Monitoring Blood Pressure (SMBP) provided immediate feedback to patients and may stimulate them to become active participants in self-care and improve adherence to medication [72]. Some interventions tried to motivate patients to recognize the monitoring of blood glucose as a tool in self-care to attain a better quality of life [39] and increase the rate of glycemic target achievement [73, 74].

**Fig. 4:**
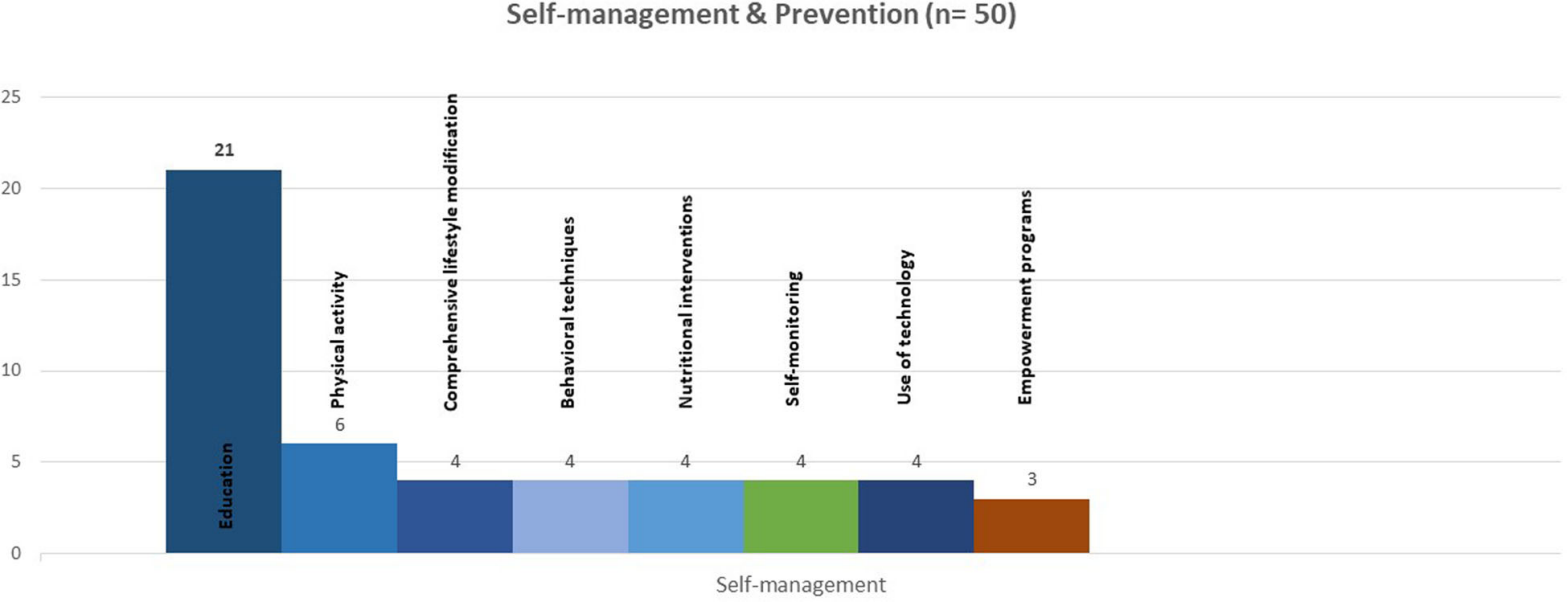
Strategies of self-management interventions

A few papers described specific adaptations made to the individuals included in the studies including tailoring to the patient’s condition [75, 76] as well as the development of a “Virtual environment” for the deaf patients with DM and HTN [77]. Technology based interventions were reported in four studies [77–80], where two interventions were delivered by nurses to deaf patients using virtual environment, offering eight screens about feeding containing food pictures and videos in Brazilian sign language, and another utilizing nurse run persuasive SMS intervention to bring about behavior change among patients. While de Souza et al, reported use of flipcharts by nurses and physical education teachers to promote quality of life and treatment adherence [72]. Bobrow et al., tested the effectiveness of non-health related messages at six-weekly intervals to hypertensive patients [80]. The different strategies of self-management interventions are described in Fig. 4.

### Continuity and coordination

The strategy of *continuity and coordination* formed the basis of eight interventions to ensure effective continuity of care for the patients. This strategy involves effective transition from one setting to another by ensuring that high quality information is conveyed between healthcare professionals during transition [24–27]. These strategies entailed auditing of healthcare data, [81, 82] referrals to specialists for specialist care, as well as behavioral persuasion approaches using letters and messages to ensure attendance and adherence [81, 82]. Only one study reported use of a multipronged approach of financial incentivization, behavioral contracting and reminder letters to retain patients in care for HTN and DM [83]. Other studies used reminder phone calls or mobile text messages to increase follow-up rates [84–86] or improve adherence [87]. The messages discussed a range of issues regarding adherence to treatment. Using this approach coupled with counselling sessions, Long et al. [86] , aimed to improve depressive symptomatology as well as glycemic indices. Three studies used clinical audits for people with HTN and/or DM in India [81], Botswana [88] and South Africa [89]. Patient referrals for specialist eye care to ensure compliance with eye control among diabetic patients was used in only one study [90].

### Information system

There were five studies that leveraged information systems [91–95], spanning use of clinical and computerized decision support systems, tele-health initiatives and use of social media. Clinical and computerized Decision Support System (DSS) developed in India and Brazil contributed to improved management of hypertensive/diabetic patients at PHC level [91, 92]. All participating professionals were trained by the research team. The decision support system implemented by Maia et al [92] in Brazil, generated treatment recommendations by entering clinical characteristics and blood glucose measurements.

Two studies focused on tele-health interventions namely: “Mobilicare” and “Telehealth Brazil” [93, 95]. The “Telehealth Brazil” program provided telemedicine services (web conference and tele-consulting) for all members of Family Health Teams (doctors, nurses and Community Health Agents) providing primary care services in remote areas to increase adherence to treatment of their HTN patients, during a six-months period [95]. Tele-consulting between professionals: through web-conferences and electronic forms (store-and-forward) with max. 48-hour reply sought to address the problem of distance, the delay in getting a second opinion, and the lack of access to it [95]. The Mobilicare service offered guidance and monitoring of diabetic patients in real time by health-care provider from a distance over one year [93]. It utilized several therapeutic approaches for instance, persuasive reminders to complete 5000 steps, as well as guidance and monitoring by health providers by using data transmitted via patient operated tablet and online glucometer. An integrated tele-health intervention delivered via Facebook group “Diabetes Macedonia” leveraged patients and caregivers education along with tailored treatment plans including pump setting, basal bolus insulin delivered via skype using the data uploaded by patients [94].

### Leadership & incentivization

Six papers involved encouraging quality in healthcare organizations through *leadership* and incentivization. All of these studies focused on teaching and training of primary care physicians (n= 5) or medical students (n= 1).

Five studies focused on training of primary care physicians that included training in the psychotherapeutic BATHE (Background, Affect, Trouble, Handling, Empathy) technique [96], Continuing Medical Education (CME) programs [97], training in regional DM management guidelines [98], and use of active teaching skills [99]. In contrast to other programs, the BATHE technique aimed at improving affect management, problem solving, and adherence among patients to improve clinical outcomes. While rest of the interventions included CME or training programs aimed at family doctors/GPs aimed to exert a positive effect on the medical decision-making process and subsequently on patient health outcomes by reducing the probability of having uncontrolled BP [97]. The only educational intervention aimed at medical students tested a curriculum pertaining to Continuity of Care Clinic designed for final year medical students, and aimed to improve their cardiovascular risk management skills during their clerkship in Thai community hospitals [100].

### Organization and equipping of healthcare teams

Organization and equipping of healthcare teams is an important strategy explored by a total of 78 interventions. This strategy involved improving the capacity of healthcare teams and organizations by supplying them with necessary medical and laboratory equipment, essential medicines to manage chronic conditions and teaching specials skills and knowledge to healthcare teams [24–27]. These interventions revolved around themes of healthcare delivery models (n= 38), screening efforts (n= 10), equipping health centers with capacities in auditing of patient records (n= 6), health system management (n= 3), exploration of mediators and moderators of healthcare delivery (n= 2), integration of health services (n= 6), and education (n= 4), lab testing (n= 2), establishment of mobile clinics (n= 2), and providing opportunities for self-management by free provision of BP monitoring services to patients (n= 2) and teaching meditation (n= 1), and equipping centers with technologies aiding in health care delivery (n= 2).

A total of six audits (after 2010), in urban areas of different regions, were conducted either by family physicians [101] or multidisciplinary teams [88, 89, 102–104]. Govender et al., conducted a doctor or nurse led audit of 40 community health centers in South Africa after providing training workshops [89]. While all the audits were based on patient records, only one study [103] conducted auditing of qualitative interviews of doctors and nurses to assess quality of healthcare services. Two studies provided training in and implementation of structured clinical records [89, 102], family physician led audit of patients pre and post implementation of guidelines, and implementation and measurement of DM care by using DM quality indicator set developed by the National Diabetes Quality Improvement Alliance [104]. Apart from audits, effect modifiers of quality of health service was explored as cross-sectional surveys in Chengdu, China and urban areas of Argentina, further leading to design and implementation of informed guidelines [105, 106].

Prevention efforts were done in using different strategies including screening campaigns, provision of health education at healthcare setups and community centers. These were conducted in rural areas of Vietnam using personal medical records [107], in rural Sudan by nursing students [108], CHW led foot screening program in South Africa [109], in Kenya using HIV counsellors trained in screening techniques and referral protocols [110]; pharmacist led screening programs in Thailand [111, 112], CHW led Behvarzes and Qazvi Health Plan in Iran [113, 114]. These efforts also included development and validity testing of risk scoring systems including Achutha Menon Center risk score in rural Kerala, India [115] and FINDRISC page questionnaire in Europe [116] based on variables like BMI, waist circumference, physical activity, dietary intake, personal and family history of HTN and high blood glucose.

Three studies explored health management systems including a Chronic Disease Outreach Program based on the Chronic Care Model in South Africa that trained 186 nurses in detection, follow up of patients with DM and HTN and referrals to specialists [117]. An NGO based healthcare delivery service in rural Guatemala was explored by Flood et al. [118], while a Kosovo based family medicine service was adapted from Dartmouth Medical School’s clinical micro-systems utilizing different interventional elements of screening, auditing of medical records and implementation of clinical guidelines, both for doctors and nurses [119].

Integrated healthcare models were explored in a total of six studies, where all except one nutritionist led intervention [120] was delivered by interdisciplinary teams [75, 121–124]. Only one of the studies provided integrated health services in a slum by a team of physicians, nutritionists, adherence counsellors, social workers, health educators in Kenyan outpatient clinics that served 1465 patients in a span of two years [121]. In China, a nutritionist led care program was tested in collaboration with physicians and dietitians [120]. Rest of the interventions provided a mix of education, skills training for health management, one to one either face to face or via skype or group-based consultations along with pamphlets and educational materials.

Provision of affordable lab testing was explored in two studies; where one utilized an oral glucose tolerance test in Thai primary care centers [125] and the second involved free lab testing for DM among 300 patients in West Bank [126]. Establishment of mobile clinics (n= 2) were tested in Sudan, run by an internist with interest in DM, an ophthalmologist, a DM nurse, DM educator and a lab technologist [127]; another study provided details on a mobile unit testing nurse run and counsellor supported mobile unit providing integrated counselling and treatment for HIV along with additional screening of tuberculosis, DM, and HTN in South Africa [128]. Self-management by free provision of BP monitoring services to patients was tested in two interventions [129, 130]. Technology based task shifting approaches were utilized in two studies [131, 132], which tested BP tele-monitoring services ensuring transmission of data from home to clinical web portals [132] while trained technician run mobile fundal cameras under supervision of ophthalmic nurse for screening of DM related retinal complication [131].

**Fig. 5:**
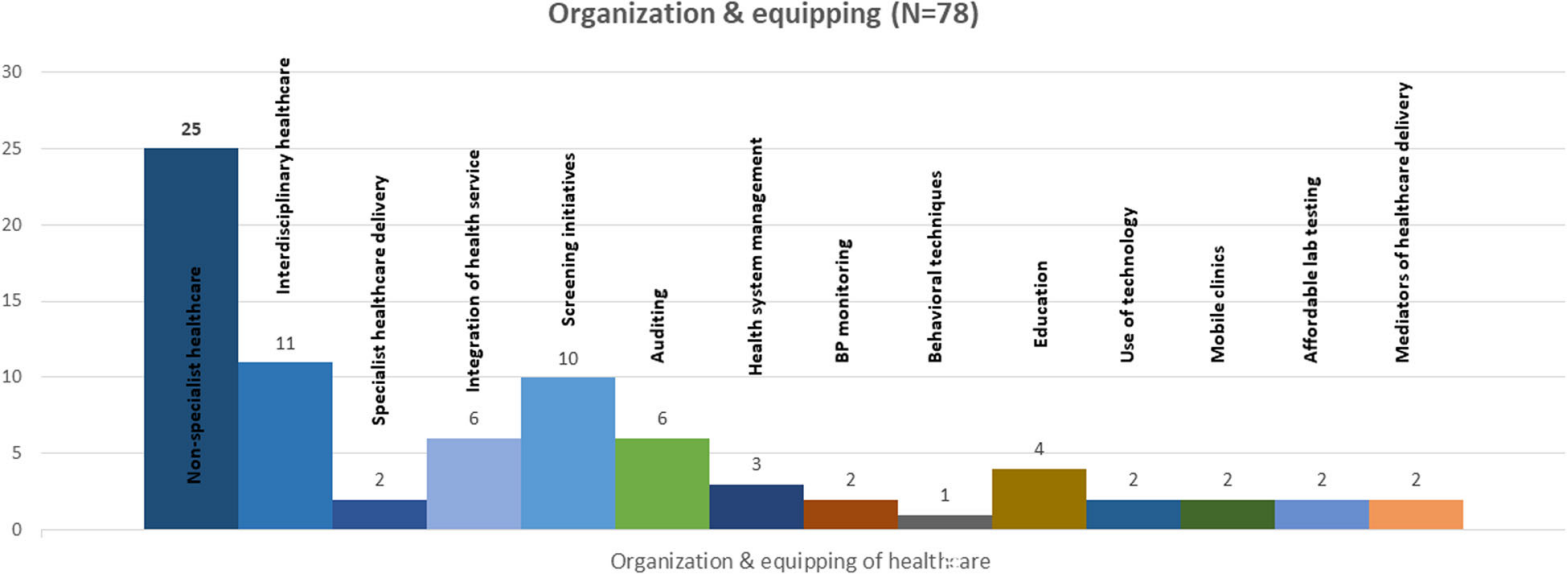
Organization and equipping of healthcare teams' elements

Only one of the studies reported a Tai-Chi based meditative intervention implemented by experienced trainers in Chinese regions of Changshu and Fangshan [133]. While educating human resource was a target in 4 interventions, promoting self-management and education integrated with regular healthcare activities in Filipino government health units [134], and integrated group sessions delivered by a trained multidisciplinary team in China [135]. Two interventions focused on training of CHWs in delivering DM prevention education [136, 137], with Gagliardino et al. delivering an educational intervention to both physicians and patients by a team of diabetologist as well as trained educators [137]. Lastly, Susliparat designed an educational module tailored for DM related complications during Ramadan, safe fasting and dose adjustments, delivered by a team of physicians and local religious leaders [138]. The different elements of the organization and equipping of healthcare teams described in the included studies are represented in Fig. 5.

A total of 25 studies focused on healthcare delivery models delivered by non-physicians. Ten of these programs focused on pharmaceutical care. Mainly, testing and individualized and group follow-up counselling sessions were integrated with regular medical care [76, 139–144]. Three pharmacist delivered interventions were unique involving risk prediction using validated tools and self-check fasting blood glucose levels [145], while rest of the interventions involved auditing of physician issued prescriptions to spot errors in dosage and drug interactions and patient records to design HTN guidelines.

Task shifting initiatives to non-specialists were explored in a number of studies on CHW based healthcare delivery models, for delivering screening, home based education, healthcare assessment as well as lab testing. CHWs were used in a quality Point Of Care Testing program [146] and providing health education and assessment of clinical indicators with or without supervision of primary care physicians [147, 148]. Another intervention explored trained local healthcare workers to deliver screenings, vouchers for free treatment to improve care seeking behavior in a slum in Nairobi [149].

Nurse led initiatives were highlighted in six interventions. These projects included POCT based HbA1c testing services, development of nursing service system as part of integrated disease management model, training of nurses in a management tool to allow nurses to prescribe restricted medications to manage DM and HTN. In three studies nurses were trained in task shifting strategies including multidisciplinary care and development of clear and standardized protocols and guidelines under supervision of a physician [150–152]. Task shifting to trained non-physician staff was tested in a number of studies. Several strategies were tested including non-physician clinicians delivered integrated management for HTN and DM [153]; nutritionists delivered medical nutrition therapy model to prescribe insulin to pregnant women [154]; public health students mediated quality assessment of integrated care delivery models [155]; and pharmacy technician delivered screening and referrals to specialists were tested [156].

Implementation and evaluation of multidisciplinary healthcare team-based health care delivery was explored in healthcare facilities (n= 9) and community setting (n= 2). These multidisciplinary teams comprised of heterogeneous healthcare professionals. A total of ten of these teams included primary care doctors followed by nurses (n= 6), dentists (n= 3), dietitians (n= 1), educators (n= 4), pharmacists (n= 2), lab technicians (n= 3), podiatrists (n= 1), specialists (n= 2), social workers (n= 1), nutritionists (n= 1), social workers (n= 1), psychologists (n= 1), physical trainers (n= 1), lay health workers (n= 4). Two of these studies involved home consultations by a multidisciplinary team that evaluated medical prescriptions [140, 157]. Early detection of DM related complications was a target for specialist doctor led screening campaigns for instance retinal complications [158] while another study tested an interdisciplinary model of assistance by multiple specialties in Brazil [159]. WHO CVD risk management package [160]; free access to spontaneous demands in treatment of HTN [161]; holistic healthcare packages [162]; DM education and smoking prevention [163] and counseling groups [164] were tested in single studies.

### Multifaceted strategies within healthcare service organization

Four studies (one focusing on HTN, all others on both HTN and DM), involved multiple aspects of the ICCCF. All these strategies involved Organization and equipping of healthcare teams through control and detection of HTN and/or DM [165–167], and creating/ strengthening of multidisciplinary Chronic Disease Management (CDM) team [168]. Other most common components were leadership through CME [165, 166], and training the multidisciplinary team [168], and Self-management through patients’ empowerment [166, 167] and health promotion [165]. Information systems were common component of two interventions with introduction of electronic medical records [165] and Chronic Disease Information [166]. Continuity/coordination was identified as continuous monitoring of medicine supply [166] (Table 1).

**Table 1:** Summary of multifaceted strategies within “Healthcare Service Organization” level

Study Details	Disease	Self -management	Continuity/ coordination	Information systems	Leadership	Org. & equip. teams
Borja-Aburto, 2016 [165]	HTN, DM	X		X	X	X
Ramli, 2014 [168]	HTN, DM				X	X
Tapia, 2016 [166]	HTN, DM	X	X	X	X	X
Tienthavorn, 2015 [167]	HTN	X				X

### Community

This strategy involved health improvement initiatives taken at the level of communities by improving their levels of awareness, mobilization and provision of leadership support. Nineteen studies (9.7%) involved strategies that targeted community level awareness (n= 4), providing leadership support (n= 3) and mobilizing communities (n= 2), while a total of ten programs were multifaceted. Four papers emphasized *raising awareness* by driving educational campaigns delivered by lay health workers [163, 169–171]. These awareness drives were conducted at primary healthcare centers, schools, workplaces, and community dwellings comprising of educational sessions on lifestyle modification as well as print media, videos and radio talks. Sahli et al. [170], described a healthy lifestyle promotion drive conducted by a team of physicians, paramedics, nutritionists and psychologists in Tunisia. Singha-dong et al., tested effectiveness of nursing students led interventions who facilitated home visits and focused group discussions [169]. A community-based volunteer led intervention [163] sought to increase awareness on HTN in the population by frequent monitoring of BP and individualized counseling on life-style modifications. One of the interventions involved posters and flyers with practical or standardized educational messages displayed in public places to alert communities about DM in children, diabetic ketoacidosis and importance of a healthy life-style [171].

Only two papers reported on *mobilizing and coordinating* [172, 173]. In many resource-poor communities with lack of healthcare professionals, community–based non-health workers could be mobilized as replacement for simple tasks as obtaining BP reading using electronic devices [173], as well as diabetic patients with good glycemic control could be engaged as peer-supporters upon completion of the necessary training program [172].

A total of three studies reported on *leadership and support* in Cameroon, where the shortage of specialized personnel being among the most important led to implementation of nurse-led protocol-driven care for HTN [174] and type 2 DM [175] at a PHC level and setting-up nurse-led pilot clinics for the management of four NCDs (HTN, Diabetes, asthma and epilepsy) at PHCs [176].

### Multifaceted strategies within community level initiatives

Ten strategies involved several components of the ICCC “Community” level: Mobilize / coordinate (n= 6); Raise awareness (n= 7); Complementary services (n= 3); Leadership and support (n= 9).

Leadership and support was a key common component, through training of the nurse educator [177] and training of the teams [148, 178–184] on the disease (s) management, assessment and education techniques with emphasis on self-management support. Initiatives to Raise awareness used CHWs for education of communities on type 2 DM and/or HTN [148, 178, 180, 182, 184, 185] or exercise clubs and health-parks [181]. These non-specialists were fluent in local languages to educate the villagers [177–179, 181, 183, 185]. Mobilizing and coordination of community members was fundamental for health activities in the communities. Complementary services were identified in 3 strategies: development of flip-charts to facilitate CHWs teaching [180], appointments and travel vouchers to a local health facility [185] and weekly club meetings and home visits if necessary [184] (Table 2).

**Table 2:** Summary of multifaceted strategies within “Community” level

Study detail	Disease	Mobilize / coordinate	Raise awareness	Complem. services	Leadership & support
Balagopal, 2008 [178]	DM	X	X		X
Bui, 2014 [177]	DM	X			X
Chamie, 2012 [179]	Both	X			X
dePue, 2013 [180]	DM		X	X	X
Getpreechaswas, 2007 [181]	HTN	X	X		X
Jafar, 2010 [148]	HTN		X		X
Jawa, 2016 [182]	DM		X		X
Khabala, 2015 [183]	Both	X			X
Kotwani, 2014 [185]	HTN	X	X	X	
Micikas, 2015 [184]	DM		X	X	X

### Policy

Seven (3.6%) papers addressed policy –level strategies including policy efforts for integration of risk factor surveillance in public-private primary care enterprises (n= 1), support for legislation (n= 3), and advocacy efforts (n= 3). Only one study aimed to improve public-private partnership model by *integrating* risk factor surveillance into the primary healthcare system in Iran using the WHO‘s STEPwise approach to NCD surveillance [186].

Three papers reported on *legislative support* in the form of institutional support, government led CHW based healthcare initiative and implementation of universal health coverage. Supportive legislation framework enabled implementation of primary HCWs led standardized HTN management in China [187], certification of National Program for improving Access and Quality in Primary Health Care (PMAQ) in Brazil by quality evaluation of 16,960 family health team coordinators [188] and improved access to care and Diabetes policy in Thailand by implementing social security scheme and universal health coverage [189]. In China, primary HCWs run HTN program was termed as the gate-keepers in healthcare where hypertensive patients > 35 years old were provided free health services including health files, annual checkups and follow-ups four times a year.

Three papers addressed *leadership* /media *advocacy efforts* for increasing knowledge and attitudes and preventive practices among the population in Vietnam, Turkey and Jamaica*.* Communication campaigns have been used to influence attitudes and behaviors of individuals to a variety of subjects including health. Mass media campaigns “Eat Less Salt” and “12/8” achieved community-based salt reduction behavior change in Vietnam [190] and increased awareness, knowledge and treatment of HTN in Turkey [191]. Port-of-Spain Declaration “Uniting to Stop the Epidemic of CNCDs in the Caribbean“ triggered the Caribbean Wellness Day (CWD), public-private and civil society partnership in health promotion in 20 countries of Caribbean Community (CARICOM) [192].

### Interventions involving Multiple Domains of ICCC Framework

Twenty one papers described strategies that covered significantly elements from multiple domains of Healthcare Service Organization, Community and Policy levels. Most frequently utilized elements of interventions were organization and equipping healthcare teams, followed by self-management, raising awareness, leadership/advocacy, support legislation, mobilize/coordinate, leadership incentives, provision of complementary services, leadership and support, policy integration and consistent financing (Table 3).

**Table 3:** Summary of Multiple Domain strategies

Study details	Disease	Healthcare Service Organization					Community				Policy			
		SM	CC	IS	L	OET	MC	RA	CS	LS	IP	SL	LA	CF
Aikins et al., 2014 [193]	Both					X	X					X		
Ameh et al. 2017 [194]	Both					X					X			
Debussche et al. 2010 [195]	DM	X				X				X				
Farzadfar et al. 2012 [196]	Both					X		X						
Geissler at al. 2015 [197]	Both	X				X								X
Gessler et al. 2012 [198]	Both	X				X		X						
Gunathilake et al. 2009 [119]	DM	X			X								X	
Hatun et al. 2015 [200]	DM					X						X	X	
Hendriks et al. 2015 [201]	Both					X						X		
Hu et al. 2010 [202]	Both					X		X						
Kamath et al. 2014 [203]	Both					X		X						
Nguyen et al. 2012 [204]	HTN					X							X	
Nugmanova, 2008 [205]	HTN					X		X					X	
Qiao, 2010 [206]	DM	X											X	
Salazar, 2014 [207]	Both	X				X			X					
Schmidt, 2011 [208]	Both					X						X		
Van Olmen, 2015 [209]	DM					X		X						
West-Pollak, 2014 [210]	DM		X				X							
Wu, 2016 [211]	Both					X							X	
Zhong, 2015 [28]	DM	X					X							
Hendriks et al. 2014 [212]	HTN					X								X
		7	1	0	1	17	3	6	1	1	1	4	6	2

The most common component of all these strategies was organization and equipping of healthcare teams to perform screenings for Diabetes and/or HTN [195, 197, 198, 208, 209, 212], to establish new approach of healthcare [193, 194, 202–204, 207], to use new guidelines and treatment protocols [201, 205, 211], to implement Diabetes program at schools [200], and to improve diabetes and HTN management [196]. Another strong common component at the level of health service organization was Self-management through health education on HTN and/or diabetes or CVD risks [28, 195, 197–199], health promotion [206] and healthy eating and physical activity education [207].

Initiatives to raise awareness used HCWs to provide communities with health education on diabetes [196, 198, 209, 202], campaigns and awareness instructions on the importance of BP checking and Coronary Heart Disease [203, 205]. Mobilizing and coordination of community health nurses was crucial in health promotion and educational programs [193], of lay community leaders in Life Style Modification (LSM) program [210], and of peer-leaders in Peer – Leader-Support – Program (PLSP) [28].

At the Policy level, Leadership and advocacy including using the media for the promotion of attitudes and health promotion campaigns [199, 204, 206], publicity about new model of health services or National Drug Benefit Package [205, 211], and National guidelines for transition of diabetic children to adult clinics [194]. Supportive legislation provided health promotion policy [209], community-based health – insurance [201], Education guidelines for T1D patients [200], and inclusion of some HTN and Diabetes drugs on pay exemption list [193]. Consistent financing strategies were used to create Universal Package of Services by Loans Fees [197] and to build a Community- based Insurance Program for hypertensive persons [212]

Remaining components of the presented strategies (Continuity /coordination and Leadership at the level of Health Service Organization, Complementary services and Leadership and support at the Community level, Integrate policies at the Policy level) were identified only in one intervention each through the implementation of LSM program following the established chronogram [210], nurse education program with guidelines adapted to local use and decision support by staff of specialists [199], provision of free anti-hypertensive drugs and distribution of free seeds of vegetables in the communities [207], training of peer-educators by a diabetologist [195], implementation of Integrated Chronic Disease Management (ICDM) model [194] respectively (Table 3).

#### Summary of evidence

While summarizing this evidence, we identified various strategies and approaches to prevent and manage HTN and DM in LMIC. The dominant strategies tested in the interventions were equipping of healthcare teams (n=78) and self-management and prevention (n= 49), while a few studies reported policy making efforts (3.6%). In the domain of organizing and equipping healthcare teams, a majority of the studies either focused on non-physician work force (n=25) or collaborative care (n=11), while, self-management and prevention involved spreading awareness among communities (n=21) and promoting physical activity (n=6). WHO-PEN was tested in only one study despite its status as a model intervention promoted by the WHO. Other efforts such as design of effective legislative frameworks, consistent financing, policy making initiatives, committed leadership and use of innovative data driven tools were seldom tested.

## Discussion

### Summary of findings

This review identified a total of 198 studies which used different designs, approaches and delivery agents to improve the management of HTN and diabetes mellitus in LMICs and PHC. More than half of all papers identified came from 1 of 5 countries, namely Brazil, China, Thailand, Mexico or South Africa. Looking at the ICCCF more than three quarters of the included studies focused on healthcare service organization with a focus in this area on interventions with an educational component and organizing and equipping healthcare teams. Patient education on importance of healthy lifestyle and CVD risk factors (smoking, harmful use of alcohol, unhealthy nutrition and physical inactivity) was a main goal of many strategies in most of the countries. Even in the majority of multifaceted interventions, self-management support and education were key components [148, 178, 180, 182, 184, 185]. Interventions on organizing and equipping healthcare teams implemented interventions on healthcare delivery models as well as interdisciplinary teams.

Overall this review highlights the diversity of possible interventions at PHC, but that despite the major efforts of the WHO to deliver the WHO-PEN package [213], only one of included studies [214] evaluated its effectiveness. In contrast, a high proportion of included studies explored non-specialist led interventions including lay health workers, peers, and community leaders. Due to poor health financing and lack of human resource in LMIC, non-specialists may have been sought for prevention and treatment efforts against NCDs especially HTN and DM [150–152]. These task shifting interventions have been found to be effective in other fields, for example maternal and child health and HIV/AIDS. Twenty studies describe an intervention that includes multiple elements from the ICCCF with the following being used most frequently: organization and equipping of teams, self-management and raising awareness. As described by Kruk et al., given the complexity of improving the quality of care, multiple components are needed to be addressed versus single component interventions [215]. Despite the current “hype” around the use of technology, only a few studies focused on technology-based services. Using the TIDieR checklist and guide [22] as a tool to extract data found a lack of reporting on the different variables. For example, only a few studies reported density and dosage of intervention, an important mediator of effectiveness of interventions. This lack of important information seriously limits reproducibility, implementation and scale-up of these interventions.

### Limitations

The present review is one of the first comprehensive presentations of primary care interventions for HTN and DM in LMICs. Despite its strengths pertaining to a broad scope of presented information, there are several limitations. The authors conducted electronic search of only three databases that may have reduced number of included articles. Moreover, no regional databases were searched. Although it was generally observed that most of the studies presented methodological biases, the review does not enlist methodological biases in included studies. A majority of included studies had descriptive and pre-post designs, based on small sample sizes and limited in geographical scope. Moreover, these studies lacked information related to implementation process such as density of dosage, fidelity rating, cost-effectiveness and training of delivery agents, therefore limiting their potential for replication in other settings. Lastly, our review is limited in scope as we have not conducted any meta-analysis assessing effectiveness of these studies.

#### Recommendations for future work

We did not take into account the scale of the interventions tested or implemented at micro, meso or macro levels. Future studies are encouraged to include this classification. In describing studies many elements of the TIDIER were not included thus limiting possible lessons learnt. For this it would be recommended that journals ensure that all publications presenting results from interventions use this framework in their reporting [216]. Given the need for multi-component complex interventions, study designs and evaluation techniques will need to be adapted by including process evaluations [217], versus simply effectiveness or outcome evaluations. Beyond the evaluation there is also the need to add new theories, such as Normalization Process Theory [218] to ensure that the intervention can later be embedded and integrated into the existing system. This will require changes in the approaches researchers undertake and in research funding in order to truly impact the delivery of care [219].

## Conclusion

Only 198 articles were found over a 10 year period which demonstrates the limited published research on highly prevalent DM and HTN in LMIC. This study shows the variety and complexity of approaches that have been tested to address HTN and DM at PHC, community and policy level. It highlights the different elements of interventions need to be addressed in order to strengthen the delivery of care. The included studies showed that multi-component interventions working at various levels of community, health service organization and policy making were generally more successful than single component studies.

## Supplementary information

**Additional file 1.** Search strategy. The complete search strategies on the different databases.

**Additional file 2.** Study characteristics. Detailed characteristics of the included studies.

**Additional file 3.** Study description and classification. Description of the included studies according to the TIDieR template and their classification according to the ICCF framework.

## Data Availability

All of the data supporting the findings of this review are included in this published article and its additional files.

## Abbreviations

CC: Continuity/Coordination
CHW: Community Health Worker
CF: Consistent Financing
CME: Continuing Medical Education
CS: Complementary Services
CVD: cardiovascular diseases
DM: Diabetes mellitus
HTN: hypertension
ICCCF: Innovative Care for Chronic Conditions Framework
IS: Information Systems
IP: Integrate Policies
L: Leadership
LA: Leadership and Advocacy
LMICs: low- and middle-income countries
LS: Leadership and Support
MC: Mobilize/Coordinate
NCD: noncommunicable diseases
OET: Organization and Equipping of healthcare Teams
PEN: Package of Essential Non-communicable Disease Interventions
PHC: primary health care
RA: Raise Awareness
RCT: randomized controlled trial
SL: Support Legislation
SM: Self Management
TIDieR: Template for Intervention Description and Replication
WHO: world health organization
