# Supplementary material for: Interventions targeting hypertension and diabetes mellitus at community and primary healthcare level in low- and middle-income countries:a scoping review
Source: BMC Public Health. 2019 Nov 21;19:1542. doi: 10.1186/s12889-019-7842-6 (PMC6873661; doi:10.1186/s12889-019-7842-6)
Supplement: Supplementary file 1 — Additional file 1. Search strategy. The complete search strategies on the different databases. [file 12889_2019_7842_MOESM1_ESM.docx]

**Annex 1 : search strategy**

1. **Pubmed:**

(Intervention or impact or effectiveness or efficacy or treatment or management or program or project or "Health Care Facilities, Manpower, and Services"[Mesh] or "Health Resources"[Mesh] or "Policy"[Mesh] or “ policy” or "Organization and Administration"[Mesh] or "Analytical, Diagnostic and Therapeutic Techniques and Equipment Category"[Mesh] or "Information Systems"[Mesh] or educational or peer or "Peer Group"[Mesh] or"Psychosocial Support Systems"[Mesh] or "Counseling"[Mesh] or Counseling or "Information Dissemination"[Mesh]) and ("Developing Countries"[Mesh] or Afghanistan or Albania or Algeria or "American Samoa"[Mesh] or “American Samoa” or Angola or Argentina or Armenia or Azerbaijan or Bangladesh or Belarus or Belize or Benin or Bhutan or Bolivia or "Bosnia and Herzegovina"[Mesh] or “Bosnia and Herzegovina” or Botswana or Brazil or Bulgaria or Burkina Faso or Burundi or Cabo Verde or Cambodia or Cameroon or "Central African Republic"[Mesh] or “Central African Republic” or Chad or China or Colombia or Comoros or Congo or Costa Rica or "Cote d'Ivoire"[Mesh] or “Cote d'Ivoire” or “ivory coast” or Cuba or Djibouti or Dominica or "Dominican Republic"[Mesh] or “Dominican Republic” or Ecuador or Egypt or El Salvador or Guinea or Eritrea or Ethiopia or Fiji or Gabon or Gambia or Georgia or Ghana or Grenada or Guatemala or Guinea or Guinea-Bissau or Guyana or Haiti or Honduras or India or Indonesia or Iran or Iraq or Jamaica or Jordan or Kazakhstan or Kenya or Kiribati or Korea or Kosovo or "Kyrgyzstan"[Mesh] or “Kyrgyz Republic” or Kyrgyzstan or Laos or Lebanon or Lesotho or Liberia or Libya or Macedonia or Madagascar or Malawi or Malaysia or Maldives or Mali or "Micronesia"[Mesh] or “Marshall Islands” or Mauritania or Mauritius or Mexico or Micronesia or Moldova or Mongolia or Montenegro or Morocco or Mozambique or Myanmar or Namibia or Nepal or Nicaragua or Niger or Nigeria or Pakistan or Palau or Panama or "Papua New Guinea"[Mesh] or “Papua New Guinea” or Paraguay or Peru or Philippines or Romania or "Russia"[Mesh] or “Russian Federation” or Rwanda or Samoa or “Sao Tome and Principe” or Senegal or Serbia or Sierra Leone or "Melanesia"[Mesh] or “Solomon Islands” or Somalia or "South Africa"[Mesh] or “South Africa” or "South Sudan"[Mesh] or “South Sudan” or Sri Lanka or "Saint Lucia"[Mesh] or “Saint Lucia” or “St. Lucia” or "Saint Vincent and the Grenadines"[Mesh] or "Saint Vincent and the Grenadines" or “St. Vincent and the Grenadines” or Sudan or Suriname or Swaziland or "Syria"[Mesh] or Syria or “Syrian Arab Republic” or Tajikistan or Tanzania or Thailand or Timor-Leste or Togo or Tonga or Tunisia or Turkey or Turkmenistan or Tuvalu or Uganda or Ukraine or Uzbekistan or Vanuatu or Venezuela or Vietnam or “Palestine” or “West Bank and Gaza” or Yemen or Zambia or Zimbabwe) and ("Hypertension"[Mesh] or hypertension or "Diabetes Mellitus"[Mesh] or "Diabetes Mellitus") and ("community health services"[MeSH Terms] OR community health care[Text Word] or "primary health care"[MeSH Terms] OR primary health care[Text Word])

1. **Embase:**

(Intervention or impact or effectiveness or efficacy or treatment or management or program or project or 'health care system'/exp or 'health care delivery'/exp or 'information'/exp OR 'health education'/exp OR 'counseling'/exp or educational or peer) AND ('developing country'/exp OR afghanistan OR albania OR algeria OR 'american samoa'/exp OR 'american samoa' OR angola OR argentina OR armenia OR azerbaijan OR bangladesh OR belarus OR belize OR benin OR bhutan OR bolivia OR 'bosnia and herzegovina'/exp OR 'bosnia and herzegovina' OR botswana OR brazil OR bulgaria OR burkina AND faso OR burundi OR cabo AND verde OR cambodia OR cameroon OR 'central african republic'/exp OR 'central african republic' OR chad OR china OR colombia OR comoros OR congo OR 'Costa Rica'/exp or 'costa rica' OR 'cote d`ivoire'/exp OR 'cote d`ivoire' OR 'ivory coast' OR cuba OR djibouti OR dominica OR 'dominican republic'/exp OR 'dominican republic' OR ecuador OR egypt OR el AND salvador OR eritrea OR ethiopia OR fiji OR gabon OR gambia OR georgia OR ghana OR grenada OR guatemala OR guinea OR 'guinea bissau' OR guyana OR haiti OR honduras OR india OR indonesia OR iran OR iraq OR jamaica OR jordan OR kazakhstan OR kenya OR kiribati OR korea OR kosovo OR 'kyrgyzstan'/exp OR 'kyrgyz republic' OR kyrgyzstan OR laos OR lebanon OR lesotho OR liberia OR libya OR macedonia OR madagascar OR malawi OR malaysia OR maldives OR mali OR 'micronesia'/exp OR 'marshall islands' OR mauritania OR mauritius OR mexico OR micronesia OR moldova OR mongolia OR montenegro OR morocco OR mozambique OR myanmar OR namibia OR nepal OR nicaragua OR niger OR nigeria OR pakistan OR palau OR panama OR 'papua new guinea'/exp OR 'papua new guinea' OR paraguay OR peru OR philippines OR romania OR 'russia'/exp OR 'russian federation' OR rwanda OR samoa OR 'sao tome and principe' OR senegal OR serbia OR sierra AND leone OR 'melanesia'/exp OR 'solomon islands' OR somalia OR 'south africa'/exp OR 'south africa' OR 'south sudan'/exp OR 'south sudan' OR sri AND lanka OR 'saint lucia'/exp OR 'saint lucia' OR 'st. lucia' OR 'saint vincent and the grenadines'/exp OR 'saint vincent and the grenadines' OR 'st. vincent and the grenadines' OR sudan OR suriname OR swaziland OR 'syria'/exp OR syria OR 'syrian arab republic' OR tajikistan OR tanzania OR thailand OR 'timor leste' OR togo OR tonga OR tunisia OR turkey OR turkmenistan OR tuvalu OR uganda OR ukraine OR uzbekistan OR vanuatu OR venezuela OR vietnam OR 'palestine' OR 'west bank and gaza' OR yemen OR zambia OR zimbabwe) AND ('primary health care'/exp or "primary health care" OR 'community care'/exp or "community care") AND ('diabetes mellitus'/exp OR 'hypertension'/exp OR 'hypertension' OR 'diabetes')

1. **Web of Science**

(Health Information Systems Or educational or peer or Psychosocial or Counseling or "Information Dissemination" or “Capacity Building” or “Health Communication” or “Health Promotion” or “Health Resources” or “Health Care Quality” or Access or Delivery or “Health Services Accessibility” or “Social Marketing” or “Health Planning” or Policy? or Organization or Administration or “Capacity Building” or Management or innovation or “Pharmacy Administration” or “Professional Practice” Diagnostic or Therapeutic or “Information system?” or educational or peer or Psychosocial or Counseling or "Information Dissemination" or Intervention or impact or effectiveness or efficacy or treatment or management or program or project) and (diabetes or hypertension) and ("community health services" or “community health care” or "primary health care") and (“Developing Countries” or Afghanistan or Albania or Algeria or “American Samoa” or Angola or Argentina or Armenia or Azerbaijan or Bangladesh or Belarus or Belize or Benin or Bhutan or Bolivia or "Bosnia and Herzegovina" or Botswana or Brazil or Bulgaria or Burkina Faso or Burundi or “Cabo Verde” or Cambodia or Cameroon or "Central African Republic" or Chad or China or Colombia or Comoros or Congo or Costa Rica or “Cote d’Ivoire” or “ivory coast” or Cuba or Djibouti or Dominica or "Dominican Republic" or Ecuador or Egypt or El Salvador or Guinea or Eritrea or Ethiopia or Fiji or Gabon or Gambia or Georgia or Ghana or Grenada or Guatemala or Guinea or Guinea-Bissau or Guyana or Haiti or Honduras or India or Indonesia or Iran or Iraq or Jamaica or Jordan or Kazakhstan or Kenya or Kiribati or Korea or Kosovo or Kyrgyzstan or “Kyrgyz Republic” or Laos or Lebanon or Lesotho or Liberia or Libya or Macedonia or Madagascar or Malawi or Malaysia or Maldives or Mali or Micronesia or “Marshall Islands” or Mauritania or Mauritius or Mexico or Micronesia or Moldova or Mongolia or Montenegro or Morocco or Mozambique or Myanmar or Namibia or Nepal or Nicaragua or Niger or Nigeria or Pakistan or Palau or Panama or “Papua New Guinea” or Paraguay or Peru or Philippines or Romania or Russia or “Russian Federation” or Rwanda or Samoa or “Sao Tome and Principe” or Senegal or Serbia or Sierra Leone or Melanesia or “Solomon Islands” or Somalia or "South Africa" or "South Sudan" or Sri Lanka or “St? Lucia” or "Saint Vincent and the Grenadines" or Sudan or Suriname or Swaziland or Syria or “Syrian Arab Republic” or Tajikistan or Tanzania or Thailand or Timor-Leste or Togo or Tonga or Tunisia or Turkey or Turkmenistan or Tuvalu or Uganda or Ukraine or Uzbekistan or Vanuatu or Venezuela or Vietnam or Palestine or “West Bank and Gaza” or Yemen or Zambia or Zimbabwe)
